# Effect of Graphene Oxide Particle Size on the Enzymatic Synthesis of Polyaniline Films

**DOI:** 10.3390/mi16111287

**Published:** 2025-11-15

**Authors:** Cynthia Guerrero-Bermea, Selene Sepulveda-Guzman, Rodolfo Cruz-Silva

**Affiliations:** 1 Tecnológico Nacional de México/ITES de la Región Carbonífera, Carretera 57, Km 120, Villa de Agujita 26950, Coahuila, Mexico; 2 Facultad de Ingeniería Mecánica y Eléctrica de la Universidad Autónoma de Nuevo León, Ciudad Universitaria, San Nicolás de los Garza 66451, Nuevo León, Mexico; 3 Centro de Investigación en Química Aplicada, Boulevard. E. Reyna 140, Saltillo 25153, Coahuila, Mexico; 4 Institute for Aqua Regeneration, Shinshu University, 4-17-1 Wakasato, Nagano 380-8553, Japan

**Keywords:** conducting polymers, peroxidase, polyaniline, hybrid materials

## Abstract

In this work, the effect of aqueous dispersions of graphene oxide (GO) and nanosized graphene oxide (nGO) on the enzymatic polymerization of polyaniline (PANI) was studied. The enzymatic polymerization of PANI was carried out in aqueous medium using toluenesulfonic acid (TSA) as the dopant, horseradish peroxidase (HRP) as the catalyst, and hydrogen peroxide (H_2_O_2_) as the oxidant, using 1.0, 2.5, and 5.0 wt% of GO and nGO. The morphology of PANI-GO/nGO composites was studied by scanning electron microscopy (SEM) and transmission electron microscopy (TEM). Further characterization was performed by thermogravimetric analysis (TGA) and spectroscopic techniques such as ultraviolet–visible (UV–Vis), Fourier-transform infrared (FTIR), Raman and X-ray photoelectronics (XPS). SEM images showed that during enzymatic polymerization, PANI completely covers the GO/nGO sheets. Furthermore, physicochemical results confirmed the production of a hybrid PANI-GO/nGO material with Van der Waals-type interactions between the oxygen-based functional groups of GO and the secondary amino bond (-NH-) of PANI. Also, cyclic voltammetry experiments were carried out in situ during the polymerization of PANI-GO/nGO films. The electrochemical response of PANI-GO/nGO composites reflects two broad oxidation peaks around 300 mV and 500 mV during anodic scanning, with reversible oxidation during cathodic scanning. Classical molecular dynamics simulations were used to understand the mechanism of the composite film’s growth.

## 1. Introduction

The synthesis of conductive polymers with graphene derivatives to create new composites is widely studied due to its numerous applications such as supercapacitors [1,2], biosensing [3,4], optoelectronics [5,6], and water treatment [7,8], among others. Polyaniline (PANI) is one of the most interesting conductive polymers due to its easy synthesis, high environmental stability, and good electrical conductivity [9,10]. PANI is usually synthesized by oxidation in strong acidic media; it might have different oxidation degrees and its transition from one form to another requires an oxidation or reduction reaction [11].

On the other hand, graphene is an electrically conductive material with a 2D shape that can be obtained through chemical synthesis [12], mechanical exfoliation [13], and chemical vapor deposition [14]. Nevertheless, all these methods result in a limited quantity of bulk graphene, and there are still no methods to produce high-quality graphene in large quantities. Graphene oxide (GO) is prepared by the oxidation and exfoliation of graphite using strong chemical oxidizers [15] and is typically used as a precursor of a graphene-like material called reduced graphene oxide. GO has a thin-sheet shape with many functional groups such as carboxyl, hydroxyl, and epoxy, which makes it water dispersible; however, these functional groups can be reduced to achieve a graphene-like material that is electrically conductive [16]. In this context, the lateral size of GO sheets is a critical parameter that modulates the reactivity and properties of GO-based composites, so its control and characterization are essential for the rational design of materials and applications [17,18]. Usually, smaller GO sheets have a higher proportion of edges and defects, which increases the density of reactive oxygenated sites and favors chemical and physical interactions with polymeric matrices, biomolecules, and functionalizing agents, therefore improving its dispersion and interfacial adhesion in nanocomposites [19]. On the other hand, GO sheets with very large size might be difficult to disperse and usually require additional treatments such as fragmentation and stabilization [20].

The synthesis of PANI and GO nanocomposites has been developed using various methods, mainly chemical aniline oxidation [21,22]. Enzymatic oxidative polymerization of aniline is an environmentally friendly alternative method of aniline polymerization [23], with the main advantages being that it is a biosynthetic method that utilizes plant-derived enzymes and is classified as ecological, recyclable, and a non-toxic catalyst. This method is well suited to be combined with GO, since due to its functional groups, it has been reported that GO acts as an enzyme immobilizer [24,25], which is an advantage because it improves the stability, catalytic activity, and reusability of enzymes.

On the other hand, it has been recently reported that PANI, mainly modified with graphene materials, shows great potential for use in water treatment due to its affinity for contaminants and low cost [7]. Different preparation and doping methods have been evaluated, along with the properties of the resulting material. In this article, we report the enzymatic polymerization of PANI in aqueous medium using toluenesulfonic acid (TSA) as the dopant, horseradish peroxidase (HRP) as the catalyst, and hydrogen peroxide (H_2_O_2_) as the oxidant, adding 1.0, 2.5, and 5.0 wt% of GO and nGO as enzyme immobilizers. Morphological and physicochemical characteristics were evaluated and compared between the two GO sizes. The growth mechanism of the composite film was studied with the aid of classical molecular dynamics simulations.

## 2. Materials and Methods

Potassium permanganate 99%, p-toluenesulfonic acid monohydrate (TSA) 98.5%, aniline 99%, and graphite flakes were purchased from Sigma-Aldrich, Toluca, Mexico. Horseradish peroxidase (HRP) 150–250 U/mg was acquired from Sigma-Life Science, St. Louis, MO, USA. Ethylenediaminetetraacetic acid (EDTA) ≥ 99.5% and Hydrogen peroxide water solution 30 wt% were procured from Fluka Analytical, Toluca, Mexico. Hydrochloric acid 37 wt% was obtained by Fermont, Monterrey, Mexico. Sulfuric acid 96.6% was supplied from CTR Scientific, Monterrey, Mexico. All reagents were of analytical grade and were used without further purification.

Graphene oxide was prepared as reported by Marcano et al. [26] with modifications, followed by prolonged sonication in the case of nGO. The polymerization reaction was carried out in a three-necked flask under a nitrogen atmosphere and with the temperature controlled at 5 °C. A total of 456 mg of TSA, 20 mL of deionized water, and 215 µL of aniline were mixed for 20 min. Subsequently, 2 mL of 1.2 wt% HRP solution at pH = 7 was added, followed by 2 mL of 3.75 wt% H_2_O_2_ with the aid of an injection pump over a period of 1 h, and then the solution was allowed to react for 2 h. In enzymatic polymerizations, TSA is used to avoid the rapid inactivation of the enzyme by hydrochloric acid. After the reaction time, the solution was dialyzed for purification. For PANI-GO/nGO composites, aliquots of 1.0, 2.5, and 5.0 wt% of GO/nGO were added before HRP and stirred for 10 min. Additionally, a PANI film and PANI-GO/nGO composite films were produced by introducing a gold-coated quartz crystal into the reactor, which was fitted to a QCM200 Quartz Crystal Microbalance from Stanford Research Systems. The film deposition was monitored using an open-circuit potential technique with an EPSILON potentiostat and a AgCl/Cl reference electrode. Table 1 shows the labels assigned to the synthesized composites.

The morphology of PANI, GO/nGO, and the synthesized composites was evaluated by SEM and TEM using FEI model Nova Nano SEM 200 and FEI model Titan G280300 equipment, Eindhoven, The Netherlands, respectively. The physicochemical properties of the obtained materials were analyzed using UV–Vis spectroscopy on a Lambda 35 PerkinElmer instrument, Shelton, CT, USA, in absorption mode. FTIR spectroscopy was conducted on a Nicolet 6700, Madison, WI, USA, in transmission mode with a KBr pellet. XPS analysis was performed using the Al K (alpha) line on an Axis-Ultra, Kratos, Manchester, UK. Raman spectroscopy was conducted with Renishaw inVia Raman equipment, Gloucestershire, UK, with two different lasers. On the other hand, thermogravimetric analyses were performed on Rigaku TGA/DTA 8120 equipment, Tokyo, Japan, at a heating rate of 10 °C/min with an air flow of 300 mL/min. Likewise, a cyclic voltammetry characterization was performed in a 0.2 N HCl monomer-free electrolyte solution using a platinum wire as the counter electrode and a 20 mV/s scan rate. Classical molecular dynamics simulations of the film’s growth were carried out using OpenMM Ver. 8.0 software and the details are provided in the Supporting Information file.

## 3. Results

### 3.1. PANI-GO/nGO Composites Preparation

**Figure 1:**
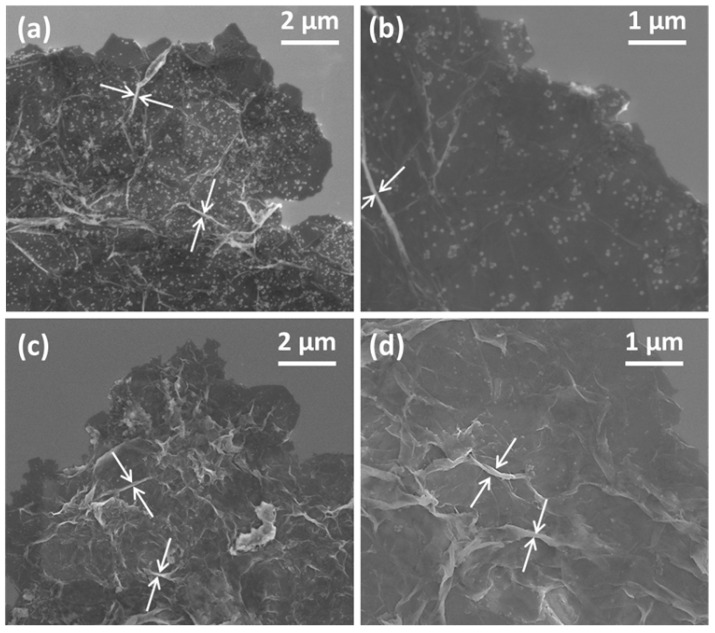
SEM micrographs of (**a**,**b**) PANI-GO2.5, and (**c**,**d**) PANI-nGO2.5. The white arrows indicate the wrinkles on the nanocomposite sheets that formed during drying.

The following results analyze the composites containing 2.5 wt% of GO and nGO, which are representative samples of the synthesized composites. Figure 1 presents the morphology of the PANI-GO2.5 (Figure 1a,b) and PANI-nGO2.5 (Figure 1c,d) composites. In all the micrographs, we can observe apparently thicker GO/nGO sheets, compared to Figure S1a,b. An increase in the thickness of the sheets is evidenced by the formation of folds (indicated by arrows), this suggests that PANI forms a thin film on the GO/nGO sheets due to the amphiphilic nature of the monomer [27]. Furthermore, mainly in the case of the PANI-GO2.5 composite, it can be observed that part of the PANI formed particle agglomerates and adhered to the surface of the GO sheets, an effect that does not occur with nGO, most likely due to the increase in surface area that the latter presents. However, one noticeable effect that occurs with nGO is that the PANI does not coat each of the nanosheets separately but instead creates a shell that encapsulates an uncontrolled amount of them.

**Figure 2:**
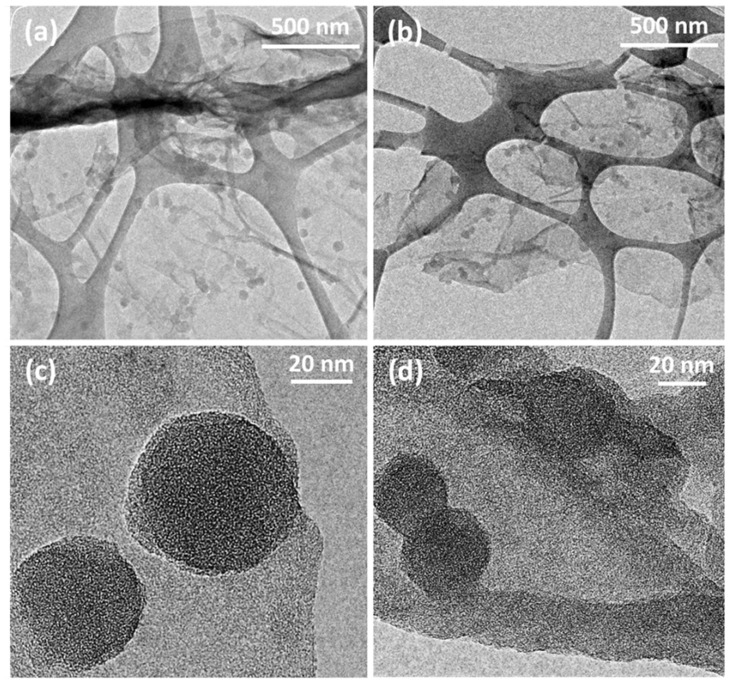
TEM micrographs of (**a**,**c**) PANI-GO2.5 and (**b**,**d**) PANI-nGO2.5, mounted on a copper grid and coated with a discontinuous silicon film.

TEM micrographs of PANI-GO2.5 (Figure 2a,c) and PANI-nGO2.5 (Figure 2b,d) are shown below. From these images, we can also assume that polymerization occurs on the GO and nGO sheets (Figure 2a,b). Furthermore, in the high-resolution TEM micrographs, the sheets show greater stability to the electron beam of the equipment, confirming that thicker sheets are obtained compared to unrecovered GO. The difference in particle size of the agglomerates seen in the SEM micrographs (Figure 1) can also be observed where the PANI-GO2.5 composite presents larger particles. Similar morphology of fully coated GO sheets with polyaniline has been obtained by chemical polymerization of aniline in the presence of GO sheets [21].

**Figure 3:**
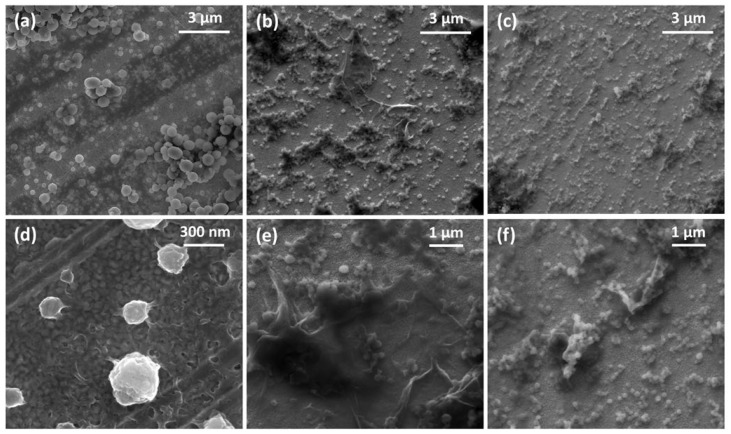
SEM micrographs of the in situ enzymatically obtained films of (**a**,**d**) PANI, (**b**,**e**) PANI-GO2.5, and (**c**,**f**) PANI-nGO2.5.

Figure 3 shows the SEM images of the films formed in situ on gold-coated quartz crystals fitted to a QCM200 Quartz Crystal balance. Figure 3a,d show the PANI film where a granular morphology with colloidal particles adhered to the surface is observed. On the other hand, Figure 3b,c show the PANI-GO2.5 and PANI-nGO2.5 films, respectively, where they are distinguished GO and nGO sheets, as well as PANI colloidal particles adhered to the graphene material. Furthermore, Figure 3d–f show a difference in size of the PANI synthesized alone and when it appears adhered to the GO and nGO sheets, where smaller adhered particles can be distinguished when nGO is used in the synthesis, as discussed in the previous morphology results.

The enzymatic polymerization reactions were simultaneously monitored by the open circuit potential (OCP) technique in the quartz crystal balance method (QCM), in order to analyze the oxidation stage and the deposition of the films by microgravimetric measurements. The evolution of the OCP during enzymatic polymerization, together with the gravimetric response and the change in the quartz crystal resistance as a function of time are graphically represented in Figure S3.

### 3.2. Composites Characterization

#### 3.2.1. Ultraviolet–Visible Spectroscopy

**Figure 4:**
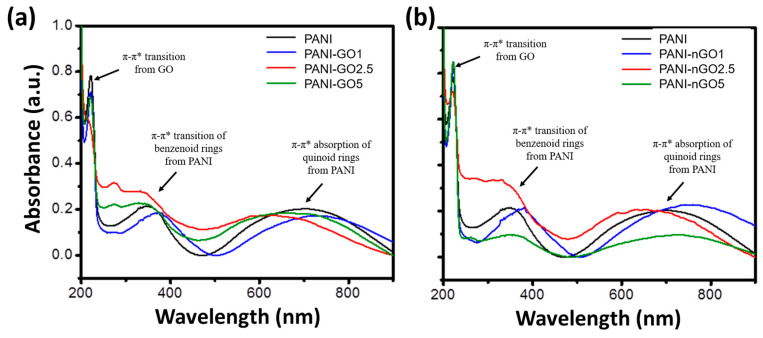
UV–Vis spectra of PANI and PANI-GO (**a**) and PANI-nGO (**b**) composites.

The UV–Vis spectra of PANI and PANI-GO/nGO composites are shown in Figure 4. The absorption spectrum of PANI (black) displays the characteristic bands of polyaniline emeraldine base formed at 320 nm and 630 nm, due to the π-π* transition of benzenoid rings and the absorption of quinoid rings, respectively [28]. On the other hand, the UV–Vis spectra of PANI-GO/nGO composites exhibit, in addition to the PANI bands just mentioned, the characteristic absorption band of GO at 230 nm, attributed to the π-π* transition [29]. Also, composite spectra experience a band shift towards the blue color. This change could be attributed to the non-covalent interactions between the functional groups of GO and those of PANI’s structure, generating hydrogen-bonding interaction >C=O∙∙∙∙H-N< [30]. This is because hydrogen bonding between PANI and GO disrupts the electronic conjugation of PANI, resulting in a reduction in the effective conjugation length.

#### 3.2.2. Fourier-Transform Infrared Spectroscopy

**Figure 5:**
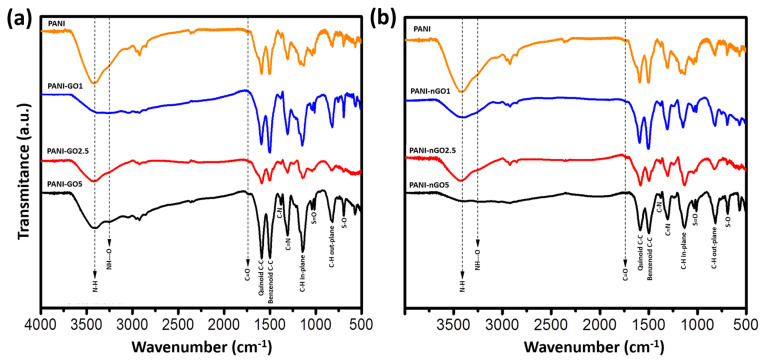
FTIR spectra of (**a**) PANI-GO and (**b**) PANI-nGO composites, compared to PANI.

The samples were analyzed by FTIR to identify the functional groups created between PANI and GO. Figure 5a shows the spectra of PANI-GO composites, while Figure 5b presents the PANI-nGO composites, in both cases compared to the spectrum of PANI. In all spectra, the predominant bands are those corresponding to the characteristic functional groups of PANI, since the highest concentration in the material corresponds to this polymer. Principal bands are located around 1599 cm^−1^, 1500 cm^−1^, 1375 cm^−1^, and 1305 cm^−1^, corresponding to the stretching vibrations of the quinoid C-C, benzenoid C-C, C-N, and C=N bonds, respectively [31]. Furthermore, in-plane bending of the C-H bond produces an absorption band at 1170 cm^−1^, whereas out-of-plane bending generates an absorption band at 830 cm^−1^ [32]. In the case of the composites, a band at 3383 cm^−1^ associated with the presence of free N-H bonds is observed, as well as a new band around 3200 cm^−1^, assigned to the formation of hydrogen bonds between PANI and the carbonyl groups of GO [33,34]. In addition, TSA residues were identified by its characteristic bands around 1100 cm^−1^ and 1030 cm^−1^, corresponding to the asymmetric and symmetric stretching vibrations of the S=O bond, and at 696 cm^−1^ for S-O stretching [35].

#### 3.2.3. X-Ray Photoelectron Spectroscopy

**Figure 6:**
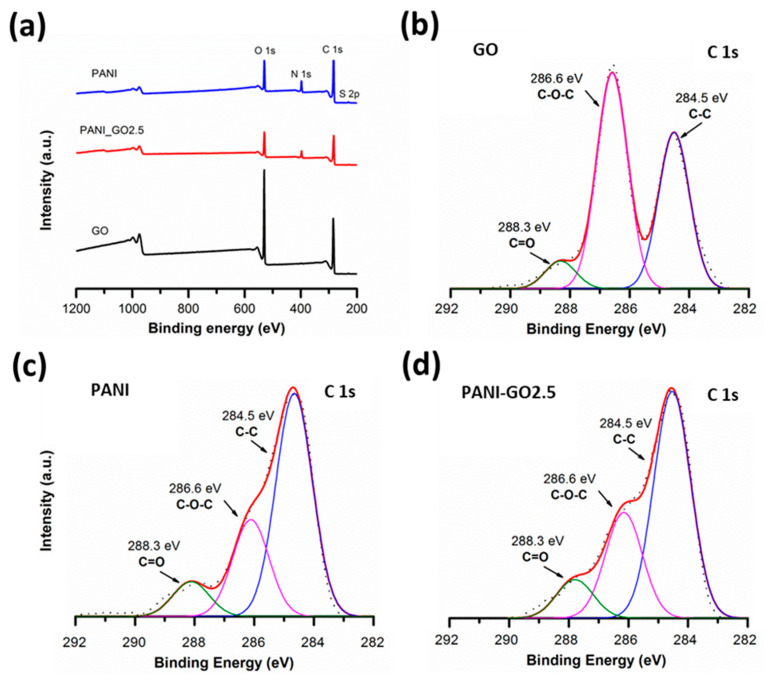
(**a**) Survey XPS spectra and high-resolution XPS spectra of C 1s peak for (**b**) GO, (**c**) PANI, and (**d**) PANI-GO2.5.

Figure 6a shows the low-resolution XPS spectra of the GO, PANI, and PANI-GO2.5 samples. It is observed that GO (black) has a carbon- and oxygen-based composition due to the photoemission peaks corresponding to O 1s around 550 eV of binding energy and C 1s between approximately 280 and 290 eV. On the other hand, PANI (blue) and PANI-GO2.5 (red) show, in addition to the C 1s and O 1s peaks, the presence of nitrogen (N 1s) and sulfur (S 2p) around 400 eV and 168 eV, respectively [34]. A higher amount of oxygen is evident for GO, having an O/C ratio of 0.48, as opposed to an O/C ratio of approximately 0.22 for PANI and the representative composite. The above reaffirms that PANI completely covers GO, otherwise the O/C ratio for the composites would be greater than 0.22. For a detailed analysis of the interaction between PANI and GO, the C 1s peak was deconvoluted. GO (Figure 6b) presents three signals, the first corresponds to the C 1s photoemission of C-C and C-H carbons in the sp2 state (284.6 eV), a second signal with greater intensity is observed at 286.5 eV corresponding to carbon with hydroxyl and oxygen groups (C-O-H and C-O-C), and finally, with a binding energy of 288 eV, there is a third signal corresponding to the carbonyl group C=O [36]. In the PANI spectrum (Figure 6c), the signals at 284.5 eV, 286.6 eV, and 288.1 eV are also identified, reported for the C-C/C-H, O-C-O/C-N, and C=O electronic environments of PANI [34]. The PANI-GO2.5 composite (Figure 6d) presents the same components as pure PANI, indicating that its structure is not modified and that the exposed surface of the composites is basically PANI.

#### 3.2.4. Raman Spectroscopy

**Figure 7:**
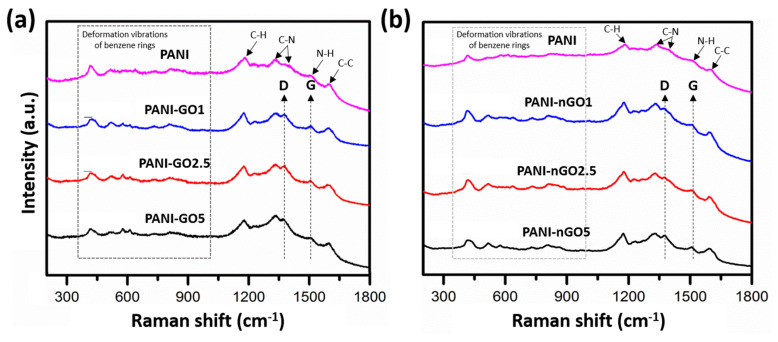
Raman spectra of (**a**) PANI-GO and (**b**) PANI-nGO composites, using moving average for elimination of spectral noise.

The Raman spectra of the PANI-GO/nGO composites are shown in Figure 7, with the spectrum of PANI as a reference. All spectra show bands from 400 to 1000 cm^−1^ related to deformation vibrations of the benzene rings, in addition to more intense bands (indicated by arrows) around 1180, 1300–1370, 1500, and 1595 cm^−1^, related to the C–H vibrations of aromatic rings, C-N stretching band of an aromatic amine, N–H deformation vibrations, and C-C stretching vibrations of the benzene ring, respectively [37,38]. On the other hand, GO is characterized by two intense peaks at 3446 and 1552 cm^−1^, explained by the presence of residual -OH groups and aromatic C=C stretching vibrations, respectively. However, the spectra of PANI-GO/nGO composites show a weak signal of these bands, which represents a reduction in GO during PANI polymerization [39].

#### 3.2.5. Thermogravimetric Analysis

**Figure 8:**
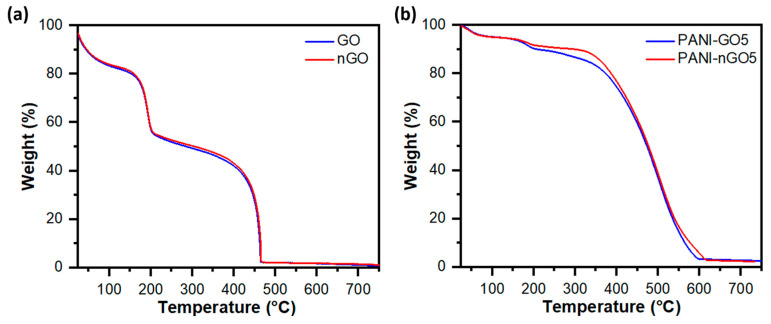
Thermogravimetric analysis of (**a**) graphene oxide samples with large (GO) and small (nGO) lateral size, and (**b**) nanocomposites of polyaniline and graphene oxide with large (PANI-GO5) and small (PANI-nGO5) lateral size.

Thermogravimetric analysis of GO with large and small sheet size and their corresponding PANI hybrids are shown in Figure 8a,b, respectively. Regardless of the sheet size, both graphene oxide samples showed similar thermal behavior. The first loss around 100 °C corresponds to bound water and the second weight loss around 200 °C to GO thermal reduction. The third and final weight loss around 450 °C is the thermally reduced graphene oxide oxidation, which leaves a very low amount of ashes. On the other hand, the thermograms of the PANI and GO composites show three weight losses, the first one below 100 °C is associated with the loss of solvents or moisture absorbed in the sample; the second loss is found between 200 and 300 °C, associated with the functional groups present in GO/nGO; the last and third weight loss is found around 400 and 600 °C, because the polymer begins to thermally degrade [40].

#### 3.2.6. Simultaneous Cyclic Voltammetry and Quartz-Crystal Microbalance Study

**Figure 9:**
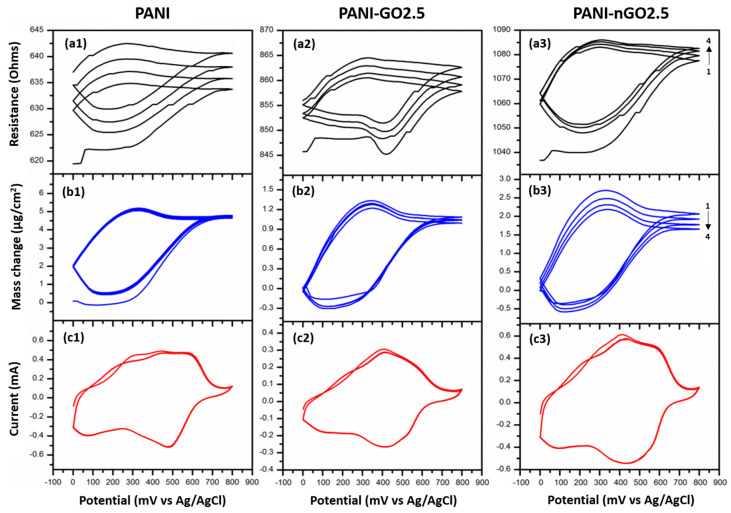
Simultaneous cyclic voltammetry/quartz-crystal microbalance characterization of PANI (**left**), PANI-GO2.5 (**middle**), and PANI-nGO2.5 (**right**) enzymatically synthesized films. (**a1–a3**) Cyclic voltammetry, (**b1–b3**) mass change, and (**c1–c3**) open circuit potential of the crystal during the enzymatic reaction.

During the polymerizations, we noticed that a thin conformal film of PANI and GO was also obtained (Figure 3). We deposited these films in gold-coated quartz-crystal and studied these films by combining cyclic voltammetry and microbalance measurements; the results are presented in Figure 9. The behavior of PANI, PANI-GO2.5, and PANI-nGO2.5 composites presents similarity, which is discussed below. The changes in resistance (Figure 9(a1–a3)) are due to changes in the energy dissipation of the quartz crystal [41,42], mainly due to changes in the damping capacity of the deposited film, which in turn result from changes in its mechanical properties. This change in the resistance of the quartz crystal is consistent with the mass changes (Figure 9(b1–b3)); both curves present the same shapes, indicating that there is no significant change in the elastic properties of the films during the test. Furthermore, the films show two broad oxidation peaks around 300 mV and 500 mV during anodic scanning (Figure 9(c1–c3)), indicating transitions between different oxidation states of PANI, specifically, from leucoemeraldine to emeraldine and from emeraldine to pernigraniline [42]. The respective reductions are shown during cathodic scanning, indicating that the oxidation is reversible. During potential scanning, the polymer becomes dedoped during the leucoemeraldine stage, reflecting a mass loss with a frequency shift (Figure 9(b1)) corresponding to a mass amount of around 4 µg/cm^2^ for PANI and higher for the composites (Figure 9(b2,b3)), due to the loss and absorption of chlorine anions during the doping–redoping process [43].

#### 3.2.7. Molecular Dynamics Simulation

**Figure 10:**
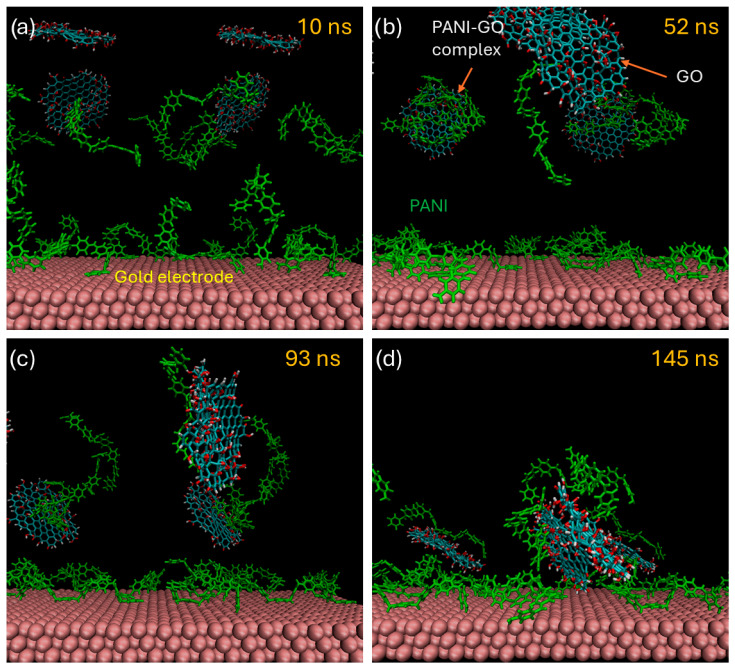
Different stages of the film’s growth as simulated by classical molecular dynamics. (**a**) Initial stage of the simulation cell with solvated GO sheets and adsorbed PANI chains on the electrode. (**b**) Formation of PANI-GO complexes, (**c**) PANI-GO complexes being dragged towards the electrode. (**d**) Irreversible adsorption of PAN-GO complex on the surface of the gold electrode coated with PANI molecules.

The mechanism of film growth was studied with the help of molecular dynamics simulations and selected frames of a simulation are shown sequentially in Figure 10. At the beginning of the simulation, the GO sheets were solvated with water molecules, while the PANI octamers rapidly adsorbed onto the gold electrode surface. These short PANI chains represent oligomers that act as nuclei for the subsequent formation of PANI precipitates. After approximately 15 ns, the PANI molecules flattened against the electrode, indicating strong interactions with the surface. The GO sheets occasionally interacted with each other through hydrogen bonding [20]; however, these contacts were weak and did not lead to irreversible aggregation. In contrast, when the PANI films interacted with the GO sheets, they established both hydrogen bonds and π–π stacking interactions between the aromatic rings of PANI and the sp^2^-rich regions of GO (Figure 10b). These interactions resulted in the irreversible formation of PANI–GO aggregates with reduced solubility. Once the PANI-GO complexes formed, the PANI molecules progressively dragged the GO sheets toward the electrode, acting as binding elements that promoted the deposition of the PANI–GO complexes onto the surface (Figure 10c,d) [37]. These simulations therefore support a mechanism of composite film formation driven by the binding ability of PANI, since GO is highly hydrophilic and alone exhibits low affinity for the gold electrode.

## 4. Conclusions

Graphene oxide (GO) is obtained by the chemical oxidation of graphite flakes, resulting in graphite oxide, which can then be exfoliated using ultrasound to produce GO nanosheets. Prolonged ultrasonic treatment of GO dispersions further fragments the sheets to nanoscale dimensions. In situ enzymatic polymerization, catalyzed by horseradish peroxidase, allows the synthesis of polyaniline–graphene oxide (PANI-GO/nGO) nanocomposites. The amount of GO used does not inhibit the catalytic activity of the enzyme during polymerization. PANI forms nanocomposites by growing directly onto the GO nanosheets, regardless of their size. Furthermore, both PANI and PANI-GO/nGO nanocomposite films are successfully deposited in situ onto gold-coated quartz substrates during the polymerization process.

## Figures and Tables

**Table 1:** Labels of PANI and GO/nGO composites.

Label	GO wt%	nGO wt%
PANI-GO1	1.0	-
PANI-GO2.5	2.5	-
PANI-GO5	5.0	-
PANI-nGO1	-	1.0
PANI-nGO2.5	-	2.5
PANI-nGO5	-	5.0

## Data Availability

The original contributions presented in this study are included in the article. Further inquiries can be directed to the corresponding author.

## Supplementary Materials

The following supporting information can be downloaded at: https://www.mdpi.com/article/10.3390/mi16111287/s1, Figure S1: SEM micrographs of (a) GO, (b) nGO, and (c) PANI; Figure S2: TEM micrographs of aqueous solutions of (a and d) GO, (b and e) nGO, and (c and f) PANI; mounted on a copper grid and coated with a discontinuous silicon film; Figure S3: Time-dependent monitoring of film formation by in situ enzymatic polymerization of polyaniline and polyaniline composites with graphene oxide. (a) Change in quartz crystal resistance, (b) gravimetric response of the quartz crystal, and (c) evolution of the open circuit potential. Figure S4: Molecular models of the polyaniline 8-mer and the nano-sized graphene oxide used in the simulations. Figure S5: Methodology used for measuring the thickness of the GO-PANI hybrids. (a) TEM image of the PANI-GO hybrid. (b) Scheme showing the morphology and orientation of the wrinkle, (c) Intensity profile used for measuring the thickness of the sheet. References [44,45,46,47,48] are cited in supplementary file.
