# Supplementary material for: Effect of Graphene Oxide Particle Size on the Enzymatic Synthesis of Polyaniline Films
Source: Micromachines (Basel). 2025 Nov 15;16(11):1287. doi: 10.3390/mi16111287 (PMC12654529; doi:10.3390/mi16111287)
Supplement: Supplementary file 1 [file micromachines-16-01287-s001.zip › micromachines-3935304-supplementary.pdf]

Supplementary Information

Effect of graphene oxide particle size on the enzymatic synthesis of polyaniline films

Cynthia Guerrero-Bermea [1], Selene Sepulveda-Guzman [2], Rodolfo Cruz-Silva [3,4,*]

[1] Tecnológico Nacional de México/ITES de la Región Carbonífera, Carretera 57, Km 120, Villa de Agujita, Coahuila, México 26950; 2157@rcarbonifera.tecnm.mx
[2] Facultad de Ingeniería Mecánica y Eléctrica de la Universidad Autónoma de Nuevo León, Ciudad Universitaria San Nicolás de los Garza, Nuevo León, México 66451; selene.sepulvedagz@uanl.edu.mx
[3] Center for Research in Applied Chemistry, Boulevard. E. Reyna 140, Saltillo, Coahuila, México 25153; rodolfo.cruz@ciqa.edu.mx
[4] Institute for Aqua Regeneration, Shinshu University, 4-17-1 Wakasato, Nagano 380–8553, Japan
* Correspondence: rodolfo.cruz@ciqa.edu.mx

Synthesis of GO, nGO, and PANI

Morphological analysis of GO, nGO, and PANI precursors was evaluated by SEM. Figures S1a and S1b show the structure of GO and nGO respectively, although chemically they are similar, the size difference between them can be clearly observed. The GO sample exhibits high exfoliation, evidenced by the transparency of the sheets and the absence of agglomerates, showing heterogeneous shape and size of up to 12.50 μm lateral distance. On the other hand, for nGO an average lateral distance of 248 nm was obtained, with greater uniformity in particle size. Also, Figure S1c shows a micrograph of PANI, presenting a uniform spherical colloidal particle structure with a diameter of approximately 70-82 nm.

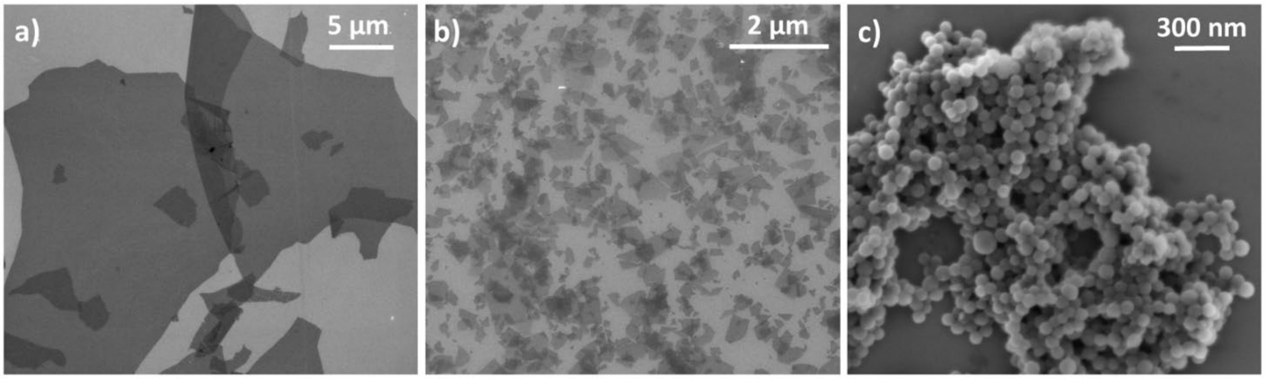

Figure S1. SEM micrographs of (a) GO, (b) nGO, and (c) PANI.

Samples were subsequently analyzed by TEM and the images are shown in Figure S2. Figure S2a, corresponding to GO, shows a sheet with certain folds (darker lines) originated by its size, a fact that is not so evident for the nGO sheet (Figure S2b). On the other hand, Figures S2d and S2e show high-resolution images where the structure of GO and nGO is analyzed, respectively. Both GO and nGO present two diffraction rings corresponding to the graphene crystalline planes $(0\bar{1}10)$ and $(0\bar{2}10)$ for interplanar distance of 0.21 nm and 0.12 nm. Furthermore, in Figures 2Sc and 2Sf the morphology of PANI can be observed, having colloids with a narrow particle diameter distribution and spherical shape, with poor colloidal stability by showing agglomerate formation.

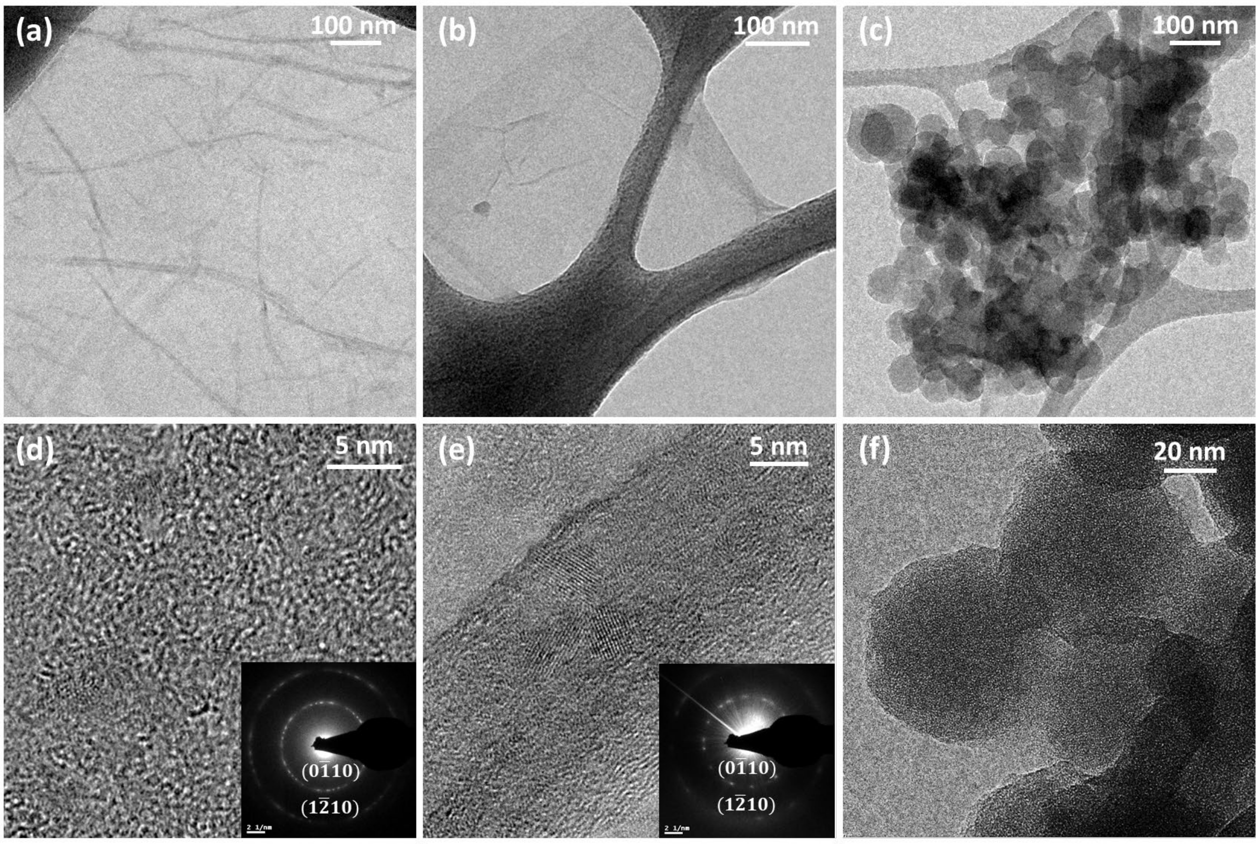

Figure S2. TEM micrographs of aqueous solutions of (a and d) GO, (b and e) nGO, and (c and f) PANI; mounted on a copper grid and coated with a discontinuous silicon film.

The enzymatic polymerization of PANI shows a mechanism where the reaction starts immediately after the first addition of hydrogen peroxide (18 min), experiencing by the rapid color change from clear to light blue. QCM was used to analyze the deposition kinetics of PANI films and PANI-GO2.5 and PANI-nGO2.5 composites during the enzymatic polymerization. The resistance represented in Figure S3a is linked to the mass change that occurs on the quartz crystal of the QCM, where there is an

induction period of the reaction just after the addition of the enzyme (minute 18) and then the oxidation of PANI continues. The induction period in the enzymatic polymerization is most likely the result of adsorption rather than autopolymerization, because enzymatic polymerization is not autocatalytic. Simultaneous observation of the frequency shift (Figure S3b) showed that there was no drastic change in the buffering capacity of the PANI film, as both curves (Figure S3a and S3b) show a similar trend. Furthermore, Figure S3c shows that once OCP increases with enzyme addition, there is a slight decrease in the working electrode, followed in some cases by increases and decreases during the first additions of hydrogen peroxide. It can be said that the decrease in the OCP is due to the hydrogen peroxide being rapidly consumed by the enzyme. As the polymerization time progresses, the OCP reaches a constant value of approximately 0.4 V, which is associated with the decrease in enzyme activity throughout the reaction.

Molecular dynamic simulations: Classical molecular dynamics (MD) simulations were performed to investigate the mechanism of film formation. The gold surface was modeled as a slab with dimensions of approximately 5.4 × 5.2 × 0.7 nm and a (111) surface orientation. Polyaniline (PANI) molecules were represented as 8-mers with a 50% oxidation degree in the salt form, carrying four positive charges along the chain. [1] To maintain charge neutrality, chloride ions were added during simulation cell assembly. The graphene oxide (GO) nanosheet was modeled as a circular sheet with a diameter of 2.4 nm. It contained an sp^2-rich region, characteristic of graphene oxide, along with a high density of oxidized functional groups—hydroxyl, epoxy, and carboxylic acid. The carboxylic groups were located at the edges of the sheet. This GO model had a molecular weight of 2,990 Da, comprising 277 atoms and 358 bonds. All molecules were parameterized using the CHARMM force field in combination with the Interface force field. [2] The simulation systems were built with the multicomponent assembler module of the CHARMM-GUI server. [3] MD trajectories were generated on a GPU workstation using OpenMM version 8.0, [4] with each simulation run for 150 ns using a timestep of 2.0 fs under NPT conditions at 30 K. Trajectories were analyzed with the VMD software. [5]

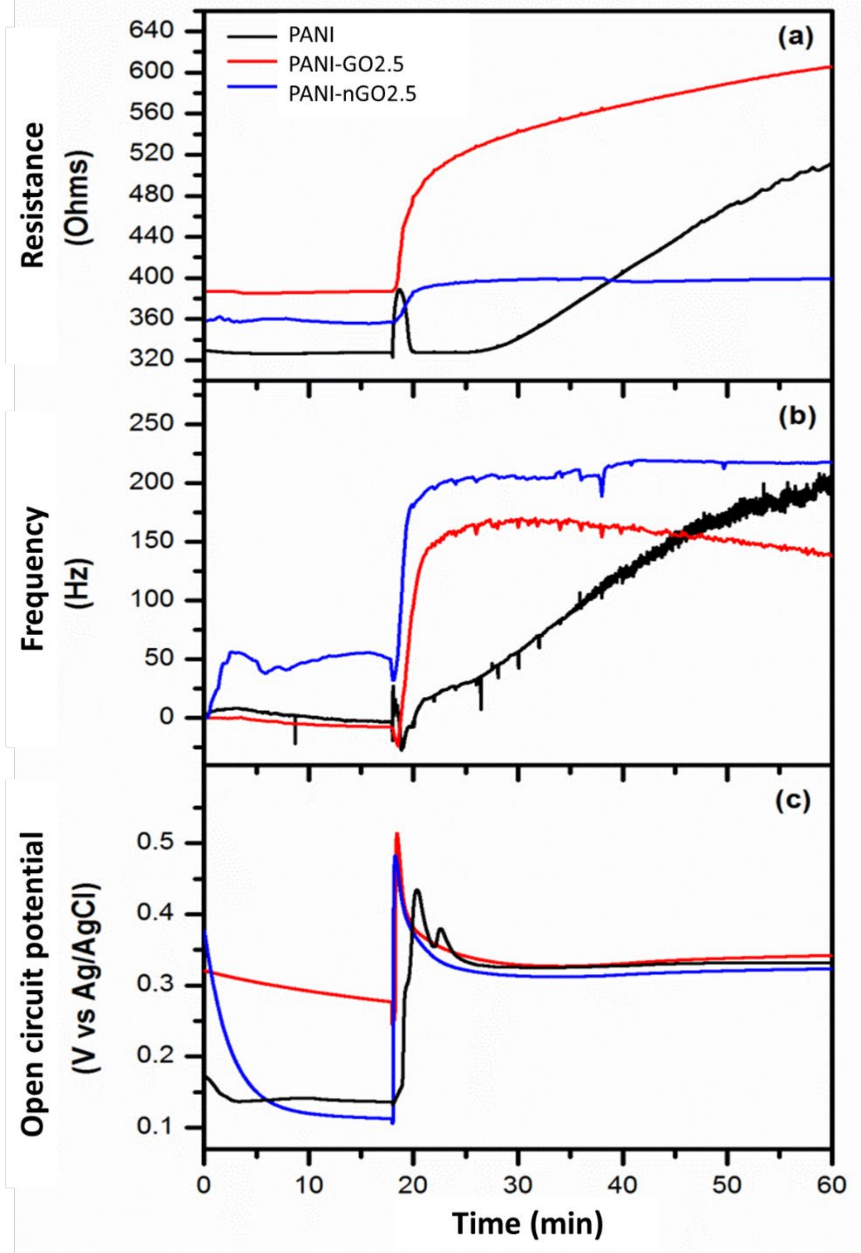

Figure S3. Time-dependent monitoring of film formation by in-situ enzymatic polymerization of polyaniline and polyaniline composites with graphene oxide. (a) Change in quartz crystal resistance, (b) gravimetric response of the quartz crystal, and (c) evolution of the open circuit potential.

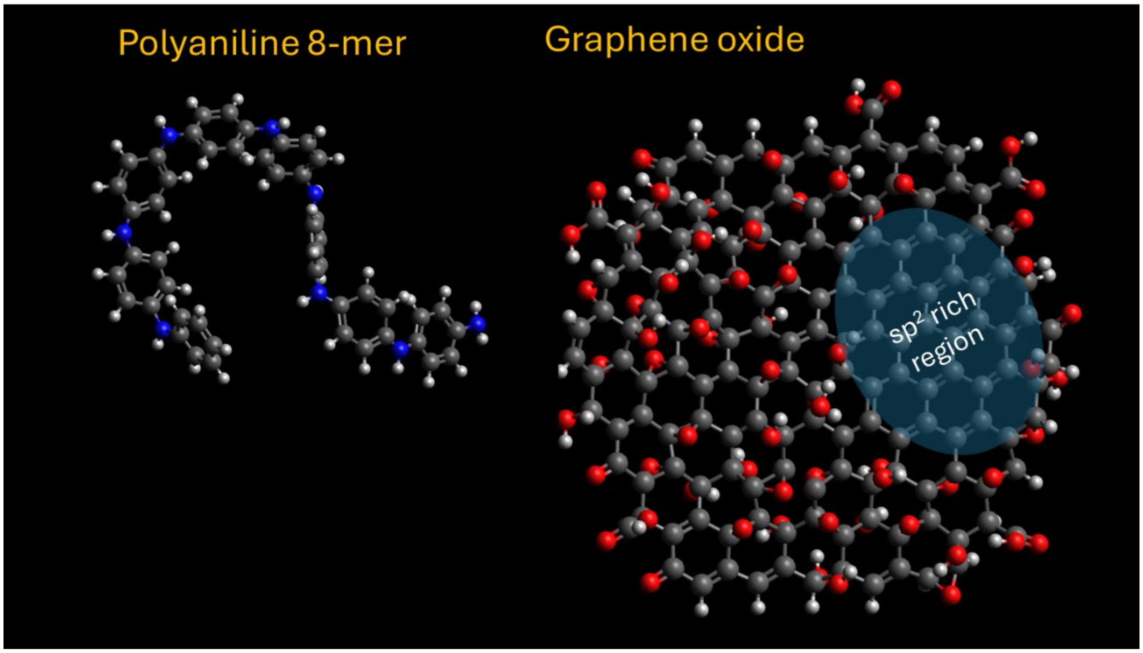

Figure S4. Molecular models of the polyaniline 8-mer and the nano-sized graphene oxide.

Analysis of the PANI-GO hybrids by TEM: The hybrids tended to exhibit small wrinkles and foldings upon drying, as shown in Figure 1. We used this feature to examine the samples by TEM (Figure S5a) and to estimate the thickness of the composite hybrids by measuring the wrinkle dimensions, as schematized in Figure S5b. After measuring the wrinkle thickness (Figure S5c), the thickness of a single-layered composite was estimated to be approximately half of the wrinkle thickness, corresponding to a value of about 9–10 nm..

Electrical conductivity measurement: PANI and PANI-GO2.5 hybrids were washed with 1% toluenesulfonic acid solution and freeze-dried to obtain the electrically conductive form. The resulting powders were compressed inside an acrylic mold to form a cylindrical pellet with a diameter of 6 mm and a thickness of 5 mm. The opposite faces of the pellet were coated with silver paint, and the electrical resistance was then measured. Bulk conductivity was Bulk conductivity σ was calculated from the formula:

$$\sigma = \frac{L}{R\,A}$$

Where R is the measured resistance, and L is the pellet thickness and A the cross-sectional area. The resulting electrical conductivity of the PANI was 0.3 S/cm and the PANI-GO2.5 was 6.5×10^{-2} S/cm.

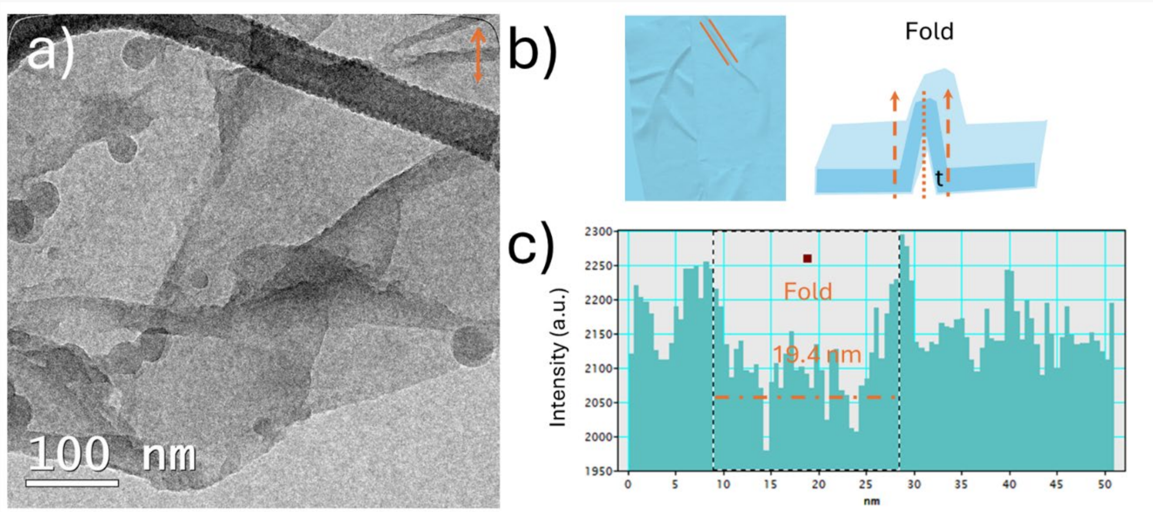

Figure S5. Methodology used for measuring the thickness of the GO-PANI hybrids. a) TEM image of the PANI-GO hybrid. b) Scheme showing the morphology and orientation of the wrinkle, c) Intensity profile used for measuring the thickness of the sheet.

References

1. Bahramian, A. Molecular dynamics simulation of surface morphology and thermodynamic properties of polyaniline nanostructured film. SURFACE AND INTERFACE ANALYSIS 2015, 47, 1-14, doi:10.1002/sia.5624.
2. Kim, S.; Lee, J.; Jo, S.; Brooks, C.; Lee, H.; Im, W. CHARMM-GUI ligand reader and modeler for CHARMM force field generation of small molecules. JOURNAL OF COMPUTATIONAL CHEMISTRY 2017, 38, 1879-1886, doi:10.1002/jcc.24829.
3. Kern, N.; Lee, J.; Choi, Y.; Im, W. CHARMM-GUI Multicomponent Assembler for modeling and simulation of complex multicomponent systems. NATURE COMMUNICATIONS 2024, 15, doi:10.1038/s41467-024-49700-4.
4. Eastman, P.; Swails, J.; Chodera, J.; McGibbon, R.; Zhao, Y.; Beauchamp, K.; Wang, L.; Simmonett, A.; Harrigan, M.; Stern, C.; et al. OpenMM 7: Rapid development of high performance algorithms for molecular dynamics. PLOS COMPUTATIONAL BIOLOGY 2017, 13, doi:10.1371/journal.pcbi.1005659.
5. Humphrey, W.; Dalke, A.; Schulten, K. VMD: Visual molecular dynamics. Journal of Molecular Graphics & Modelling 1996, 14, 33-38, doi:10.1016/0263-7855(96)00018-5.
